# Necessity of antiviral treatment for patients with chronic hepatitis B in the grey zone based on liver pathology analysis

**DOI:** 10.1080/07853890.2024.2399757

**Published:** 2024-09-16

**Authors:** Jianna Zhang, Sijie Yu, Kailu Zhu, Shibo Li, Yu Huang

**Affiliations:** a Department of Nephrology, The First Affiliated Hospital of Wenzhou Medical University, Wenzhou, China; b Department of Infectious Diseases, Zhoushan Hospital Wenzhou Medical University, Zhoushan, China; c Department of Infectious Diseases, Taizhou First People’s Hospital, Taizhou, China; d Department of Infectious Diseases, The First Affiliated Hospital of Wenzhou Medical University, Wenzhou, China

**Keywords:** Chronic hepatitis B, grey zone, Ishak, liver biopsy

## Abstract

**Objective:**

28–55% of chronic hepatitis B (CHB) patients belong to the grey zone (GZ). By analyzing the pathological characteristics of the liver of patients in the GZ, this study clarified whether the patients in the GZ need anti-hepatitis B virus treatment.

**Method:**

We reviewed 324 cases of liver pathology that underwent liver biopsy between 2011 and 2022. According to the total score of inflammation G, 0–6 points are classified as mild, 7–12 points are classified as moderate, and 13–18 points are classified as severe. According to the total score of fibrosis F, 0–2 is mild, 3–4 is moderate, and 5–6 is severe. Significant histological diseases (SHD) are defined as the presence of inflammation *G* ≥ 6 and/or fibrosis *F* ≥ 2 in liver biopsy specimens.

**Result:**

324 GZ patients were scored using the Ishak method, with G7-18 accounting for 9%, F3-6 accounting for 19.2%, and SHD accounting for 37%. The inflammation, fibrosis, and SHD in the HBeAg (+) group were more pronounced than those in the HBeAg (-) group. Among the GZ-A ∼ GZ-D subgroups, the highest proportion of SHD in the GZ-B group was 58.35%.

**Conclusion:**

More than 1/3 of the patients in GZ need anti-hepatitis B virus treatment. More than half of GZ-B patients need anti-hepatitis B virus treatment. It is very necessary to carry out rescue anti-hepatitis B virus treatment for patients in GZ as soon as possible.

## 1. Introduction

The latest version of chronic hepatitis B (CHB) treatment guidelines or guidelines [1–3] issued by the American Association for the Study of Liver Diseases (AASLD), European Association for the Study of the Liver (EASL) and Asian-Pacific Association for the Study of the Liver(APASL), according to alanine aminotransferase(ALT), HBV-DNA and hepatitis B e antigen, CHB is divided into four phases: (1) immune tolerance phase, (2) immune active phase or immune clearance phase, (3) inactive phase or low replication phase, (4) reactivity period. Such patients should generally receive antiviral treatment. After receiving antiviral treatment, the probability of developing cirrhosis and HCC in these patients significantly decreased [4].

However, clinically, the condition of patients with CHB is more complicated, about 28–55% of patients with CHB do not meet the diagnostic criteria of stage 4 [1], that is, patients cannot be attributed to any of the above 4 stages. These patients were classified as a grey zone (GZ). By the status of HBeAg, ALT and HBV DNA levels [5, 6], GZ patients into four categories: 1. Grey area A (GZ-A): HBeAg positive, the ALT level is a normal value, serum HBV DNA ≤ 10^6 IU/ml; 2. Grey zone B (GZ-B): HBeAg positive, the ALT levels are greater than the upper limit of normal, serum HBV DNA ≤ 2 × 10^4 IU/ml; 3. Grey zone C (GZ-C): HBeAg negative, the ALT levels is a normal value, Serum HBV DNA ≥ 2 × 10^3IU/ml; 4. Grey zone D (GZ-D): HBeAg negative, the ALT levels are greater than the upper limit of normal, serum HBV DNA ≤ 2 × 10^3 IU/ml. Among them, the upper limit of normal (ULN) ALT was 25 U/L for females and 35 U/L for males [1, 5].

There is little existing literature on the prevalence and natural history of this group [7, 8]. Studies have shown that if CHB patients in the GZ do not receive antiviral treatment, the probability of clinical events increases [9], and even advanced fibrosis or even cirrhosis occurs [5]. At present, there is no definitive conclusion on whether patients in the GZ should actively receive antiviral treatment or the probability of developing HCC. Therefore, it is necessary and important to evaluate the degree of inflammation and fibrosis in liver pathology for patients in GZ.

This paper attempts to focus on the statistics and analysis of liver biopsy pathology of patients in the GZ, analyze the situation of patients in the GZ from the perspective of pathology, and determine whether these patients need anti-hepatitis B virus treatment.

## 2. Methods

### 2.1. Criteria for inclusion of patients in GZ

**Figure 1.**
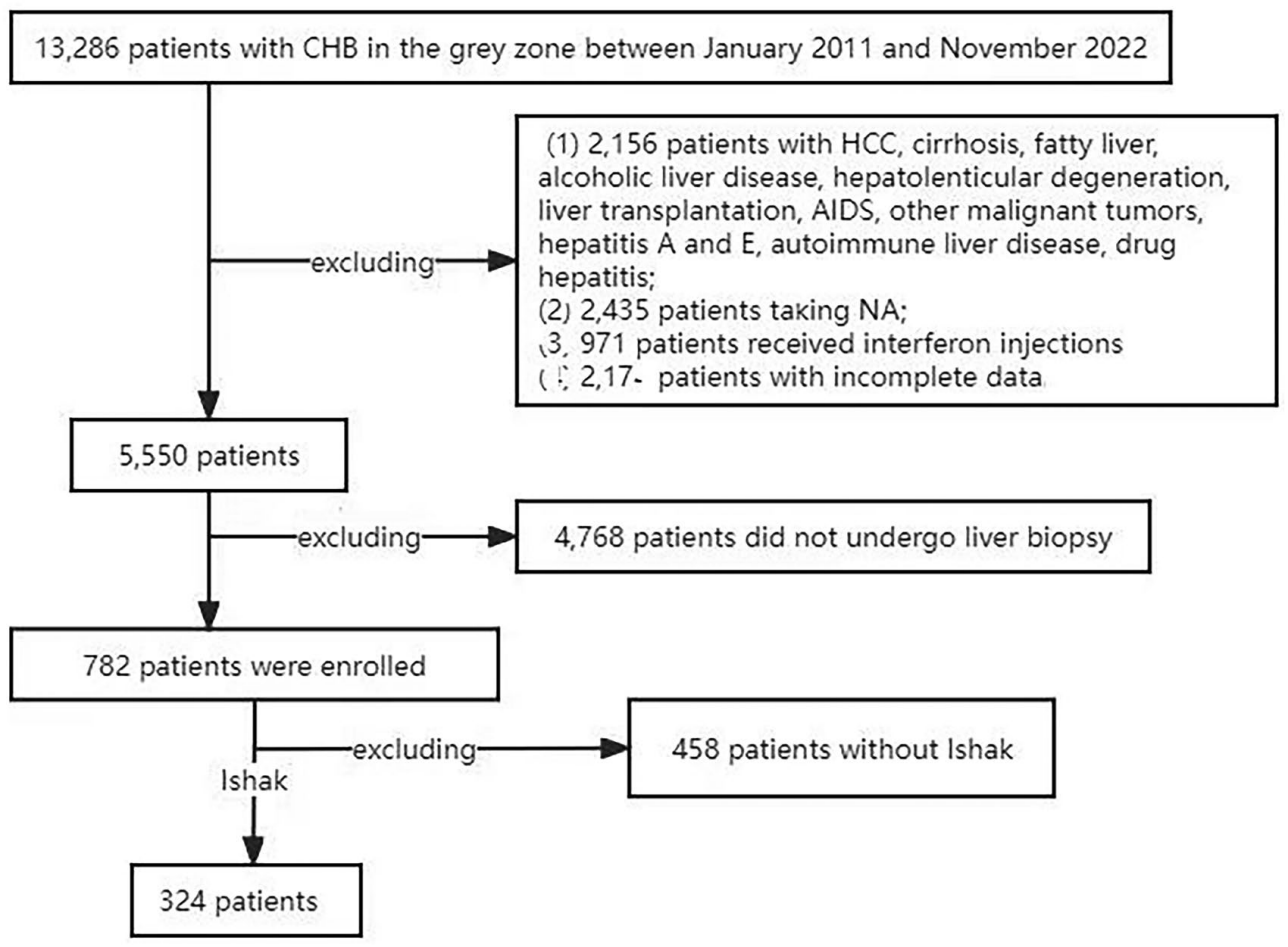
Flow chart for selecting the population for pathological study of liver biopsy in patients with CHB in the GZ.

Patients with CHB who were hospitalized at the First Affiliated Hospital of Wenzhou Medical University from January 2011 to October 2022 and did not receive antiviral treatment were included retrospectively. The diagnostic criteria for CHB was serum hepatitis B surface antigen (HBsAg) positive more than 6 months (the patient’s discharge diagnosis includes: chronic viral hepatitis B, except the discharge diagnosis of chronic hepatitis C/viral hepatitis E/viral hepatitis A/alcoholic fatty liver/nonalcoholic fatty liver disease/primary biliary cholangitis/autoimmune hepatitis/Wilson disease/cirrhosis/cirrhosis decompensated stage/liver cancer/liver malignancy/liver transplantation/AIDS/acquired immune deficiency syndrome/patients with other malignant tumors). No anti-hepatitis B virus drugs (i.e. entecavir tablets, adefovir ditexins, telbivudine tablets, tenofovir tablets, long-acting or short-acting interferon needles, etc.) were used prior to the liver biopsy pathology. A total of 324 patients with CHB who met the definition of GZ and had liver pathology were selected (Figure 1).

Ethical approval for this study had been filed in the First Affiliated Hospital of Wenzhou Medical University and approved by the ethics committee (KY2023-R001). The study process was conducted in accordance with the Declaration of Helsinki. After each patient signs a written informed consent form agreeing to undergo liver biopsy, liver biopsy examination is conducted.

### 2.2. Collection of clinical and pathological data

Clinical and pathological data of patients with CHB who met the enrollment criteria were collected in a retrospective manner. All patients underwent percutaneous hepatic liver biopsy under ultrasound localization. The 18 G biopsy needle was used in liver biopsy. Obtained liver biopsy specimens were formalin-fixed, embedded in paraffin, and stained with hematoxylin-eosin. Appropriate diagnosis of the biopsy specimen included at least six portal areas in the observation sample. Histological assessment of the liver for each liver pathology section was scored by two experienced pathologists. Based on the pathological results, to determine the patient’s liver inflammation grade and fibrosis stage.

### 2.3. Data collection and data stratification

Values for age, sex, body mass index(BMI), liver function (including ALT, AST, γ-GT, alkaline phosphatase (ALP), Albumin, platelet count (PLT), HBV-DNA, HBsAg, FIB-4, APRI, ALBI were collected. The FIB-4 index, an indicator of liver fibrosis, is defined by four factors: age, AST, ALT, and PLT.FIB-4 index = Age(year)×AST(U/L)/[PLT(×10^9/L)×ALT(U/L)]. The APRI score is AST and platelet ratio index (aspartate aminotransferase to platelet ratio index, APRI) used for the evaluation of cirrhosis, APRI calculation formula: [AST/upper limit of normal (ULN)×100]/PLT (×10^9/L). Albumin-bilirubin (ALBI) as the evaluation of liver reserve function was calculated using the linear equation: (log10 bilirubin (μmol/L)×0.66)+(albumin(g/l)× −0.085).

Data were stratified by criteria for age (<40 or ≥ 40), BMI(≥25 or <25kg/m^2^), FIB-4 (≤3.25 or >3.25) [5, 10, 11], APRI (<2 or ≥ 2) [5], and ALBI (<-2.60 or≥-2.60). The above clinical data of patients were collected within the week prior to the patient’s liver biopsy puncture and obtaining liver pathological specimens.

### 2.4. Statistics

Before conducting statistics, assess the normality of the data, manage missing data, and manage skewed data etc. Continuous variables are expressed as the median (interquartile range). The Mann-Whitney U test was used for continuous variables. For categorical variables, tested by chi-square test or Fisher’s exact test. The statistical significance is defined as *p* < 0.05. All statistical analyses were performed using Stata (version 17.0) and R language (R version 4.2.3).

## 3. Result

### 3.1. Clinical characteristics of 324 patients using Ishak system scoring GZ-A ∼ GZ-D in four subgroups

As described in Table 1, 324 GZ patients who underwent pathological scoring using the Ishak method were included. Among them, there were 75 cases in the GZ-A group, 26 cases in the GZ-B group, 132 cases in the GZ-C group, and 91 cases in the GZ-D group. The proportion of male patients in the GZ-A to GZ-D groups was higher, and there were significant differences in gender, age, and age stratification among the four groups. Due to the definition of the four groups themselves, the levels of ALT, AST, ALP, and r-GT were significantly increased in the GZ-B and GZ-D groups. The FIB-4 index showed no difference between the GZ-A to GZ-D subgroups. The FIB-4 stratification showed significant differences between the GZ-A-GZ-D subgroups (*p* = 0.020), while the APRI and APRI stratification showed significant differences between the GZ-A-GZ-D subgroups (*p* < 0.001). FIB-4 > 3.25, as an indicator for evaluating liver fibrosis, accounted for 15.4% in the GZ-B group, while APRI ≥ 2, as an indicator for evaluating liver cirrhosis, accounted for 15.4% in the GZ-B group. There was no difference in ALBI, ALBI stratification, PT, PTA, and INR among the four subgroups of GZ-A-GZ-D. The difference of hepatitis B surface antigen among the four groups was significant (*p* < 0.001).

**Table 1.** Clinical data results of 324 GZ patients using the Ishak grading score.

	GZ-A	GZ-B	GZ-C	GZ-D	*p* value
	*n* = 75	*n* = 26	*n* = 132	*n* = 91	
Gender (male, %)	53 (70.7%)	22 (84.6%)	81 (61.4%)	73 (80.2%)	0.008
Age (years)	39 (31, 47)	38.5 (29, 44)	43 (36.5, 47)	42 (36, 46)	0.020
<40	41 (54.7%)	16 (61.5%)	43 (32.6%)	35 (38.5%)	0.003
≥40	34 (45.3%)	10 (38.5%)	89 (67.4%)	56 (61.5%)	
BMI (kg/m2)	21.67 (20.2, 23.47)	22.24 (20.89, 23.29)	22.525 (20.43, 24.68)	22.94 (21.45, 24.22)	0.30
<25	65 (86.7%)	20 (77.0%)	105 (79.5%)	59 (64.8%)	0.35
≥25	10 (13.3%)	6 (33.0%)	27 (20.5%)	32 (35.2%)	
ALT (U/L)	23 (17, 29)	52.5 (38, 79)	23 (18.5, 27)	40 (30, 54)	<0.001
AST (U/L)	25 (19, 29)	38 (30, 74)	24 (21, 27)	31 (27, 40)	<0.001
ALP(U/L)	73 (61, 83)	103 (81, 130)	70 (59, 87.5)	80 (65, 101)	<0.001
γGT (U/L)	20 (14, 37)	52 (32, 132)	21 (14, 29)	33 (21, 63)	<0.001
Albumin (g/dl)	46.6 (44.1, 51.7)	46.15 (42.6, 49.2)	46.05 (43.05, 48.4)	46.7 (43.9, 51.4)	0.24
Platelet count (×10^9/L)	166 (130, 220)	168.5 (131, 205)	192.5 (168, 244.5)	181 (140, 223)	0.001
FIB-4 score	1.08 (.74, 1.95)	1.24 (.83, 2.51)	1.055 (.755, 1.445)	1.13 (.78, 1.62)	0.27
≤ 3.25	72 (96.0%)	22 (84.6%)	129 (97.7%)	88 (96.7%)	0.020
>3.25	3 (4.0%)	4 (15.4%)	3 (2.3%)	3 (3.3%)	
APRI score	.34 (.25, .46)	.57 (.36, 1.38)	.28 (.22, .385)	.39 (.29, .6)	<0.001
<2	75 (100.0%)	22 (84.6%)	132 (100.0%)	89 (97.8%)	<0.001
≥2	0 (0.0%)	4 (15.4%)	0 (0.0%)	2 (2.2%)	
ALBI score	−3.21 (-3.65, −2.98)	−3.16 (-3.43, −2.85)	−3.19 (-3.385, −2.985)	−3.21 (-3.69, −3.04)	0.19
<-2.60)	70 (93.3%)	21 (80.8%)	124 (93.9%)	86 (94.5%)	0.094
≥-2.60	5 (6.7%)	5 (19.2%)	8 (6.1%)	5 (5.5%)	
PT(s)	13.8 (13.1, 14.3)	13.75 (13.2, 14.5)	13.65 (13.2, 14.1)	13.5 (13, 14.2)	0.39
PTA (%)	92 (85, 101)	90.5 (82, 99)	93.5 (86, 100.5)	94 (86, 104)	0.38
INR	1.05 (.99, 1.11)	1.06 (1.01, 1.15)	1.04 (1, 1.095)	1.04 (.98, 1.1)	0.35
HBsAg (IU/ml)	1309.1 (832.7, 1882.6)	1215.6 (1049, 2463)	1326.7 (830.6, 1599.7)	1113.3 (556.2, 1374)	0.016
HBV-DNA	37000 (1800, 190000)	1900 (500, 5900)	9900 (4400, 31000)	500 (500, 810)	<0.001

### 3.2. Pathological analysis of Ishak grading scores in four groups of GZ-A to GZ-D in GZ

In this study, a total of 324 liver biopsy specimens from GZ patients were pathologically analyzed by Ishak grade score. In the Ishak scoring system, four parameters were introduced to evaluate the inflammation, with the highest total score of 18 points [12]. According to the total score of inflammation G, 0–6 points are classified as mild, 7–12 points are classified as moderate, and 13–18 points were classified as severe [12]. According to the total score of fibrosis F, 0–2 was mild, 3–4 was moderate, and 5–6 was severe [13]. Significant histological disease(SHD) was defined as the presence of inflammation ≥ 6 and/or fibrosis ≥ 2 in the liver biopsy specimen [14].

**Figure 2.**
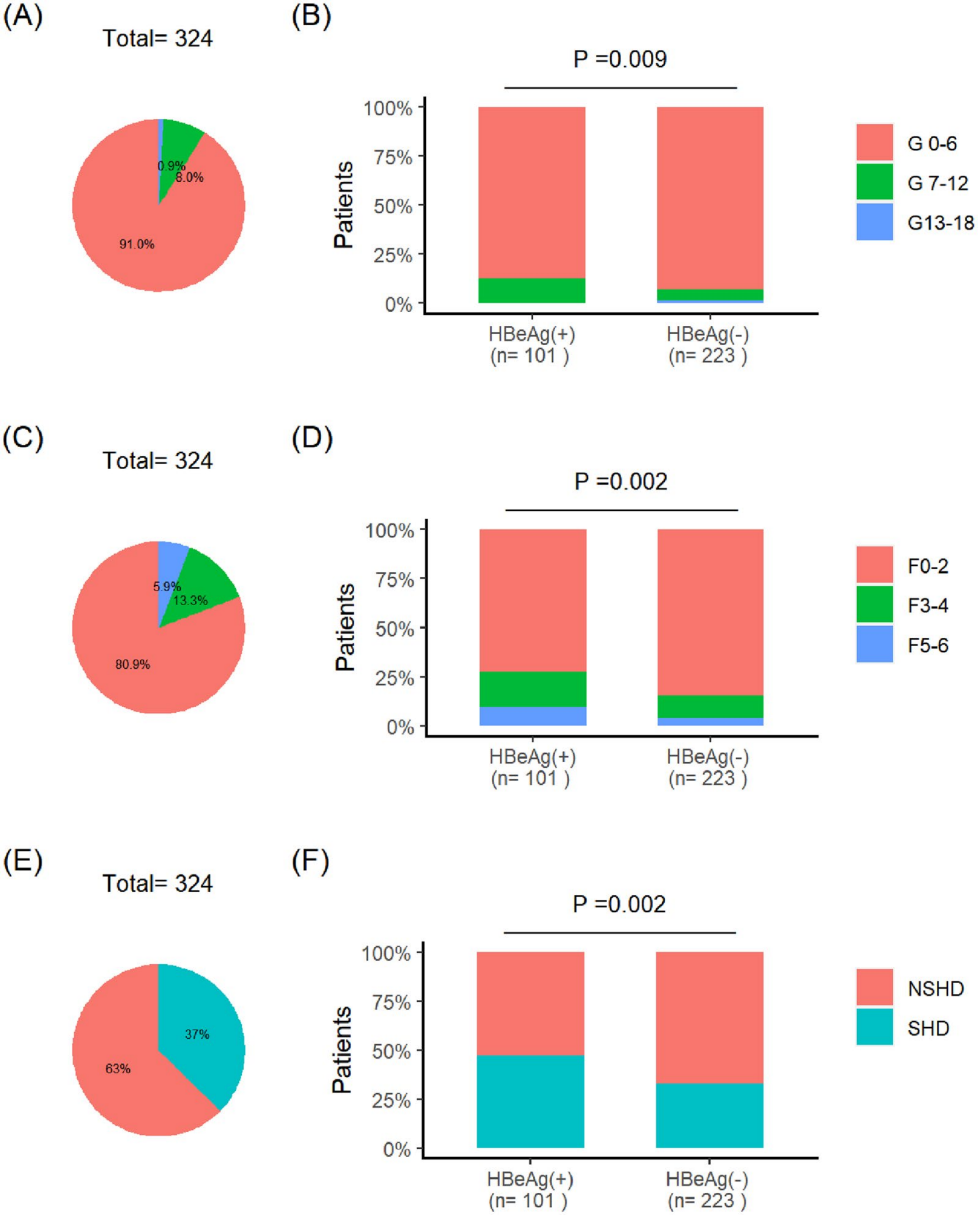
(A) 324 patients based on inflammation G grading ratio; (B) HBeAg (+) and HBeAg (-) groups based on inflammation G grading ratio; (C) 324 patients based on fiber grading ratio; (D) HBeAg (+) and HBeAg (-) groups based on fiber grading ratio; (E) 324 patients have significant histological disease(SHD) proportion; (F) the proportion of SHD in the HBeAg (+) and HBeAg (-) groups.

Among the 324 patients, the proportion of patients with G-grade being severe was 1%, moderate was 8%, and mild was 91% (Figure 2A). The proportion of HBeAg (+) group patients with moderate to severe symptoms is relatively high, accounting for 12.87%. Among them, the moderate proportion is 12.87%, and the severe proportion is 0%. The proportion of patients with moderate to severe HBeAg (-) group was 7.18%. Among them, the moderate proportion is 5.83%, and the severe proportion is 1.35% (Figure 2B). There was a significant difference in inflammation between the HBeAg (+) group and the HBeAg (-) group (*p* = 0.009). Among 324 patients, 19.2% were classified as moderate to severe according to F-grade (Figure 2C). The proportion of HBeAg (+) group patients with moderate to severe symptoms is relatively high, accounting for 27.72%. Among them, the moderate proportion is 17.82%, and the severe proportion is 9.9%. The proportion of patients in the HBeAg (-) group with moderate to severe inflammation is 15.25%. Among them, the moderate proportion is 11.21%, and the severe proportion is 4.04% (Figure 2D). Among 324 patients, 37% had SHD. In the HBeAg (+) group, about 50% of patients have SHD, which is significantly higher than that of the HBeAg (-) group, with a significant difference (*p* = 0.002) (Figure 2E). There was a significant difference in fiber grading between the HBeAg (+) group and the HBeAg (-) group (*p* = 0.002) (Figure 2F).

**Figure 3.**
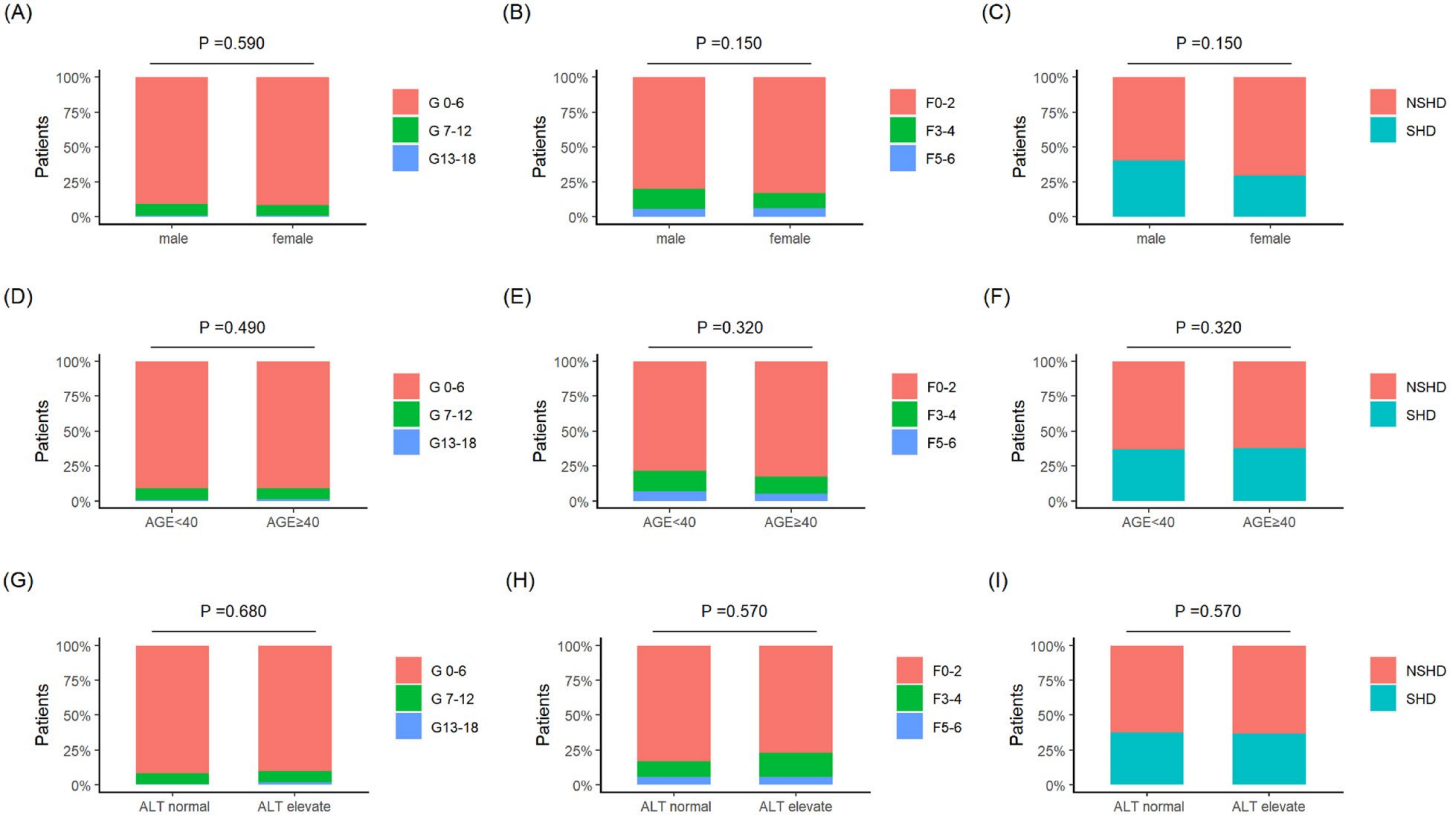
(A) Ishak inflammation grading ratio after gender grouping; (B) Ishak fiber grading ratio after gender grouping; (C) SHD ratio after gender grouping; (D) Ishak inflammation grading ratio after age stratification grouping; (E) Ishak fiber grading ratio after age stratification grouping; (F) significant histological disease ratio after age stratification grouping; (G) Ishak inflammation grading ratio after grouping based on normal and elevated ALT; (H) Ishak fiber grading ratio after grouping based on normal and elevated ALT; (I) significant histological disease proportion after grouping based on normal and elevated ALT.

According to gender, 324 patients were divided into two groups, and the inflammation G, fibrosis F, and significant histological diseases of the two groups were compared, and the results showed no difference (Figure 3A–C). Stratified by age of 40, the inflammatory G, fibrotic F, and SHD of the two groups were compared, and the results showed no difference (Figure 3D–F). 324 cases were combined with GZ-A group and GZ-C group to form the normal ALT group, while the GZ-B group and GZ-D group were combined to form the elevated ALT group. Comparing the inflammation G, fibrosis F, and SHD between the two groups, the results showed no difference (Figure 3G–I).

**Figure 4.**
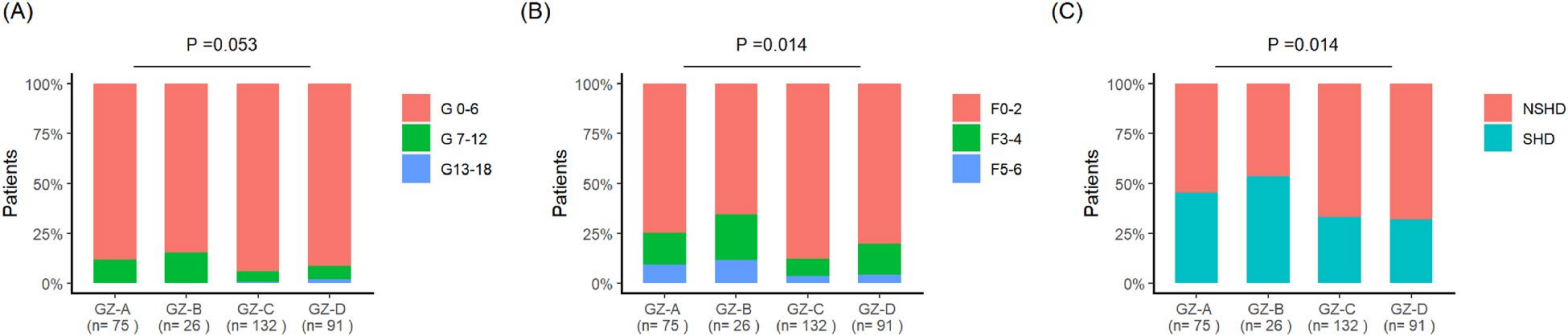
(A) Ishak inflammation grading ratio of GZ-A-GZ-D 4 subgroups; (B) Ishak fiber grading ratio of GZ-A-GZ-D 4 subgroups; (C) SHD proportion of GZ-A-GZ-D 4 subgroups.

The proportion of liver inflammation, liver fibrosis stage and SHD were analyzed between the GZ-A to GZ-D 4 subgroups. In terms of liver inflammation grading, the GZ-B group had the highest proportion of moderate to severe cases, accounting for 15.38%, followed by the GZ-A group (12%), GZ-D group (8.79%), and GZ-C group (6.06%), with no difference between the four subgroups (*p* = 0.053) (Figure 4A). Among them, the GZ-D and GZ-C subgroups had severe inflammation grading, accounting for 2.2% and 0.76% respectively. In terms of liver fiber grading, the GZ-B group had the highest proportion of severe cases, accounting for 34.62%, followed by the GZ-A group (25.33%), GZ-D group (19.78%), and GZ-C group (12.3%), with significant differences between the four subgroups (*p* = 0.014) (Figure 4B). There was a significant difference of SHD among the four subgroups (*p* = 0.014), with the highest proportion of 58.35% in the GZ-B subgroup (Figure 4C).

Considering that the GZ subgroup has a relatively high proportion of inflammation and fibrosis, as well as a significant proportion of SHD, the highest proportion in the GZ-B group was 58.35%. The correlation analysis between FIB-4/APRI and fibrosis F in patients showed correlation coefficients of 0.296 and 0.190, respectively. We screened 95 patients with inflammation G< =5 and/or fibrosis F< =1 (without SHD) and followed them up. The results showed that the median time for 11 patients to develop cirrhosis was 4.858 years (CI 2.335–7.217). Based on the pathological biopsy data and clinical data during pathological biopsy, 324 patients in the GZ-A ∼ GZ-D subgroup were followed up, and a total of 96 patients developed cirrhosis. The probabilities of liver cirrhosis in the GZ-A ∼ GZ-D subgroups in the 5th year were 11.96%, 5.13%, 4.38%, and 5.00%, respectively. The probabilities of liver cirrhosis in the 10th year were 67.50%, 76.53%, 37.91%, and 38.51%, respectively. There was no statistically significant difference in the probability of developing cirrhosis among the four groups (*p* = 0.099) (Supplementary Figure 1). The GZ-A ∼ GZ-D subgroups were compared between the two groups, and after adjustment for both unadjusted and IPTW, there was no statistically significant difference between the GZ-A ∼ GZ-D subgroups (Supplementary Figures 2–3).

## 4. Discussion

It can be seen from the current research that there are many patients with CHB clinically, which do not conform to the previous four stages of chronic hepatitis B, and these patients are named as patients in the GZ of CHB. According to the different levels of HBeAg, ALT, and HBV DNA [5, 6], patients in the GZ of CHB are classified into four subgroups: GZ-A ∼ GZ-D. 324 patients in the GZ were scored using the Ishak method, with G7-18 accounting for 9%, F3-6 accounting for 19.2% and SHD accounting for 37%. The inflammation, fibrosis, and SHD in the HBeAg (+) group were more pronounced than those in the HBeAg (−) group. Among the four subgroups of GZ-A ∼ GZ-D, the GZ-B subgroup had the highest proportion of patients with G7-18, accounting for 15.38%. In terms of liver fiber grading, the GZ-B group had the highest proportion of severe cases, accounting for 11.54%, and the GZ-B group had the highest proportion of SHD, reaching 58.35%.

This study analyzed liver biopsy specimens from our center that lasted for 12 years. The Ishak grading scoring systems were used for pathological evaluation, respectively. In the Ishak scoring system, four parameters were introduced to evaluate inflammation, namely portal area inflammation, interfacial inflammation, fusion necrosis, and intralobular activity. Hepatitis (debris like necrosis) at the interface around the portal area and fibrous septum, with a score of 0–4 points; fusion necrosis, score 0–6; Focal (punctate) lytic necrosis, apoptosis, and focal inflammation, with a score of 0–4; inflammation in the manifold area, with a score of 0–4. Calculate the inflammation values of each parameter separately and add them together, with a total score of up to 18 points [12]. The fiber score is 0–6 points based on the degree of fibrosis in the portal and central venous areas. According to the total score of inflammation G, 0–6 points are classified as mild, 7–12 points are classified as moderate, and 13–18 points were classified as severe [12]. According to the total score of fibrosis F, 0–2 was mild, 3–4 was moderate, and 5–6 was severe [13]. Significant histological disease (SHD) was defined as the presence of inflammation ≥ 6 and/or fibrosis ≥ 2 in the liver biopsy specimen [14].

Antiviral treatment for chronic HBV infection can reduce the HCC risk in CHB patients with immune active diseases [4]. The histological evaluation of the liver in CHB patients is an important indicator for initiating antiviral treatment. It is recommended that CHB patients with SHD start antiviral treatment [3]. Liver biopsy pathology is considered the gold standard for staging and grading chronic liver disease. In this study, FIB-4 > 3.25, as an evaluation index for liver fibrosis, accounted for 15.4% in the GZ-B group, and APRI ≥ 2, as an evaluation index for liver cirrhosis, accounted for 15.4% in the GZ-B group. However, liver biopsy pathology showed that the proportion of significant histological diseases in the GZ-B group reached 58.35%. It can be seen that the measurement criteria of FIB-4 > 3.25 and APRI ≥ 2 are far from meeting the clinical evaluation of GZ CHB patients. Moreover, 37% of patients in the GZ have SHD. Wang et al. [15] found that over 90% of HBeAg positive GZ CHB patients exhibited SHD. At present, there is no anti-hepatitis B virus treatment for the patients in these GZ clinically.

A follow-up study of 1465 HBV infected individuals for 6.3 years showed that the cumulative incidence of HCC in immunotolerant, active, inactive, and CHB GZ patients was 0% (0/10), 21% (96/457), 3% (3/112), and 9% (80/886), respectively. The cumulative incidence of HCC in CHB GZ patients was significantly higher than that in immunotolerant and inactive patients [16]. 1303 patients with baseline CHB GZ and 1370 patients with inactive CHB were followed up for 10 years. The cumulative incidence of HCC in the former (2.7%) was 4.5 times higher than that in the latter (0.6%). Among them, 686 patients who remained in the GZ of CHB during the 10 year follow-up period had a cumulative incidence of HCC of 4.6%; 857 patients were still inactive chronic hepatitis B patients during 10 years of follow-up, and their cumulative incidence of HCC was 0.5%, the former was 9.6 times higher than the latter [17]. At present, there are many patients in GZ, and the probability of HCC is higher than expected.

In clinical practice, FIB-4 and ARPI were used to evaluate the severity of liver diseases, especially the degree of liver fibrosis and cirrhosis, which could to some extent replace liver tissue biopsy. Our study conducted a correlation analysis between FIB-4 and ARPI with liver fibrosis F, and the results showed correlation coefficients of 0.296 and 0.190, respectively. There was a significant difference between the non-invasive evaluation indicators FIB-4, ARPI, and actual pathology, and it was not yet possible to use FIB-4 and ARPI as a substitute for liver pathology biopsy. 324 patients in the GZ-A ∼ GZ-D subgroups were followed up for 10 years, and all subgroups had a higher incidence of liver cirrhosis. In the 10th year of follow-up, the probability of liver cirrhosis in the GZ-B subgroup was even as high as 76.53%. There was no statistically significant difference in the probability of developing cirrhosis among the four groups after unadjustment and IPTW. But from the trend in the graph, it could be seen that the GZ-B subgroup had the highest probability of developing cirrhosis. There is no statistical difference between the four subgroups, which might be related to the fact that only 324 cases were reported in this study, and there might be a bias in the probability of developing cirrhosis. Further confirmation was needed. However, only 8 out of 324 patients developed liver malignant tumors. Due to the small number of endpoint cases, we did not list these data.

The follow-up results of patients in the GZ strongly support the fact that patients in the GZ are prone to developing HCC. On the other hand, the pathological results also strongly suggest that it is necessary to carry out rescue anti-hepatitis B virus treatment for more than 30% of patients in GZ, especially for CHB patients in GZ-B group, more than 50% of patients need anti-hepatitis B virus treatment. However, there is no guidance on anti-hepatitis B virus for patients in GZ in the current guidelines [1–3]. Therefore, from the above data, it is very necessary to carry out rescue anti-hepatitis B virus treatment for patients in GZ as soon as possible. However, it is still necessary to carry out liver biopsy to determine which GZ patients should receive anti-hepatitis B virus treatment. However, meaningful clinical indicators cannot be selected solely through single or multiple factor analysis and screening of existing clinical data.

This study has several limitations. Firstly, this study is a retrospective study rather than a prospective study. Due to the fact that GZ is a new concept proposed by guidelines in recent years, and liver biopsy is an invasive examination that carries the risk of causing pain, bleeding, shock and death in patients, conducting prospective liver biopsy studies on GZ patients is not an easy task. Secondly, the data in this article are all from single center clinical research data, rather than multi-center clinical research data. Thirdly, the number of patients with uncertain hepatitis and liver pathology is relatively small. Our center had been conducting liver biopsy for years, but only 324 patients who met the GZ and had pathological biopsy had been diagnosed. This is relatively new to the concept of GZ. In the past, clinical doctors often overlooked this group of patients and did not attach great importance to them. Fourth, only by analyzing and screening the existing clinical data with single or multiple factors, it is impossible to screen out meaningful clinical indicators to determine which GZ need anti-hepatitis B virus treatment. Currently, liver biopsy is still needed for clarification.

In this study, Ishak pathological scoring system was used for evaluation. The results showed that the HBeAg(+) group had more significant inflammation, more significant fibrosis and more SHD than the HBeAg(-) group. Among the four subgroups, patients in the GZ-B subgroup had the highest proportion of inflammation, fibrosis, and SHD. From the pathological point of view, the clinical gold standard confirms that patients in the GZ of CHB need to be paid enough attention and attention in the clinic, and begin to use anti-hepatitis B drugs for rescue treatment as soon as possible.

## Supplementary Material

Supplemental Material

## Data Availability

Data supporting the results of this study can be obtained from the corresponding author. Due to privacy or ethical limitations, these data cannot be publicly accessed.
