# Supplementary material for: Necessity of antiviral treatment for patients with chronic hepatitis B in the grey zone based on liver pathology analysis
Source: Ann Med. 2024 Sep 16;56(1):2399757. doi: 10.1080/07853890.2024.2399757 (PMC11407419; doi:10.1080/07853890.2024.2399757)
Supplement: Supplemental Material [file IANN_A_2399757_SM8435.zip › Suppl_Data/Supplementary Figure Caption.docx]

**Supplementary Figure 1**.The cumulative probability of liver cirrhosis in the GZ-A~GZ-D subgroups

**Supplementary Figure 2.**Comparison of two groups with cumulative liver cirrhosis in GZ-A~GZ-D subgroups

**Supplementary Figure 3.**Comparison of two groups with cumulative liver cirrhosis after IPTW adjustment in GZ-A~GZ-D subgroups
