# Supplementary figures and images for: Necessity of antiviral treatment for patients with chronic hepatitis B in the grey zone based on liver pathology analysis
Source: Ann Med. 2024 Sep 16;56(1):2399757. doi: 10.1080/07853890.2024.2399757 (PMC11407419; doi:10.1080/07853890.2024.2399757)

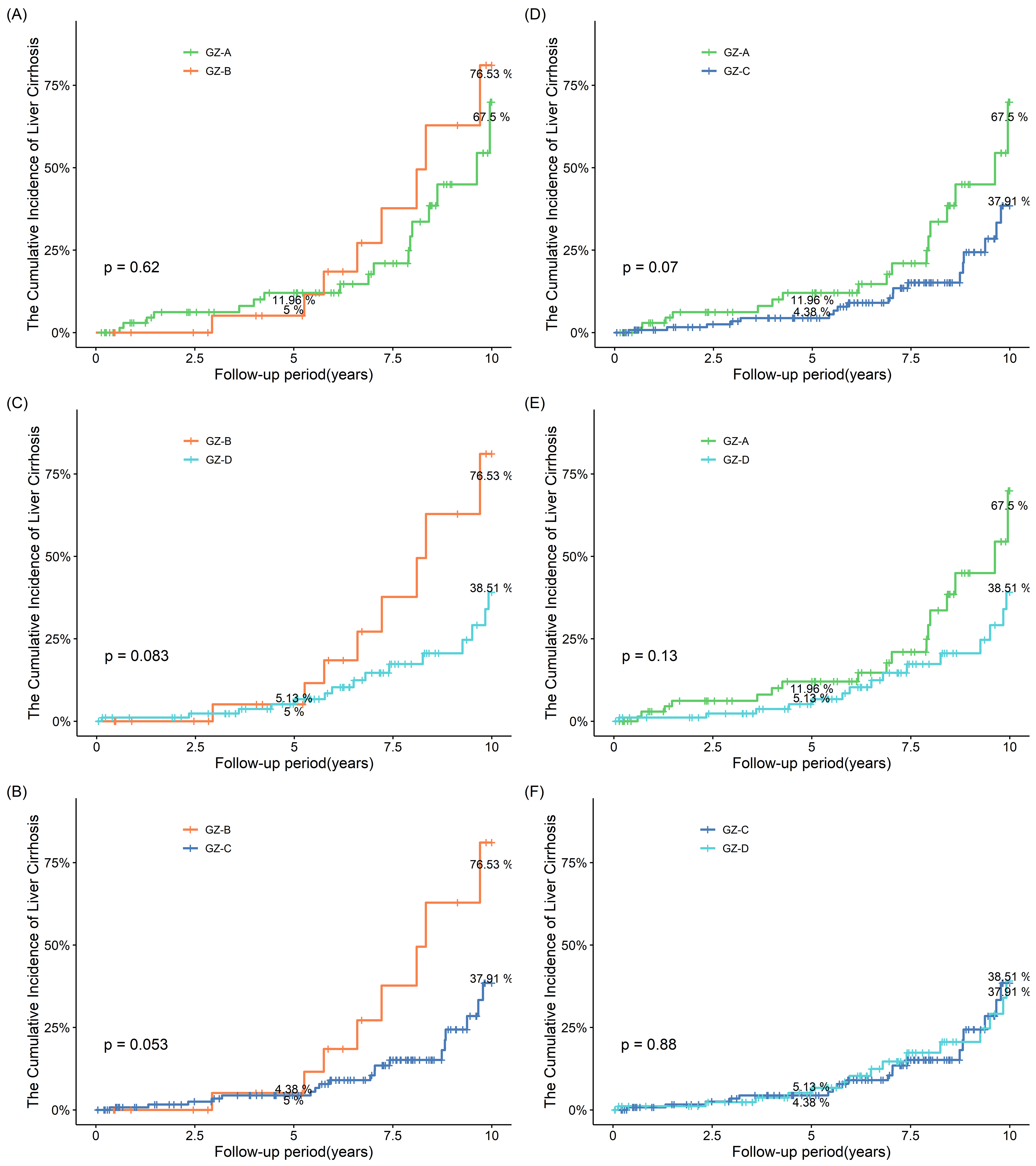

Supplement: Supplemental Material [file IANN_A_2399757_SM8435.zip › Suppl_Data/Supplementary Figure 2(300dpi).tif]

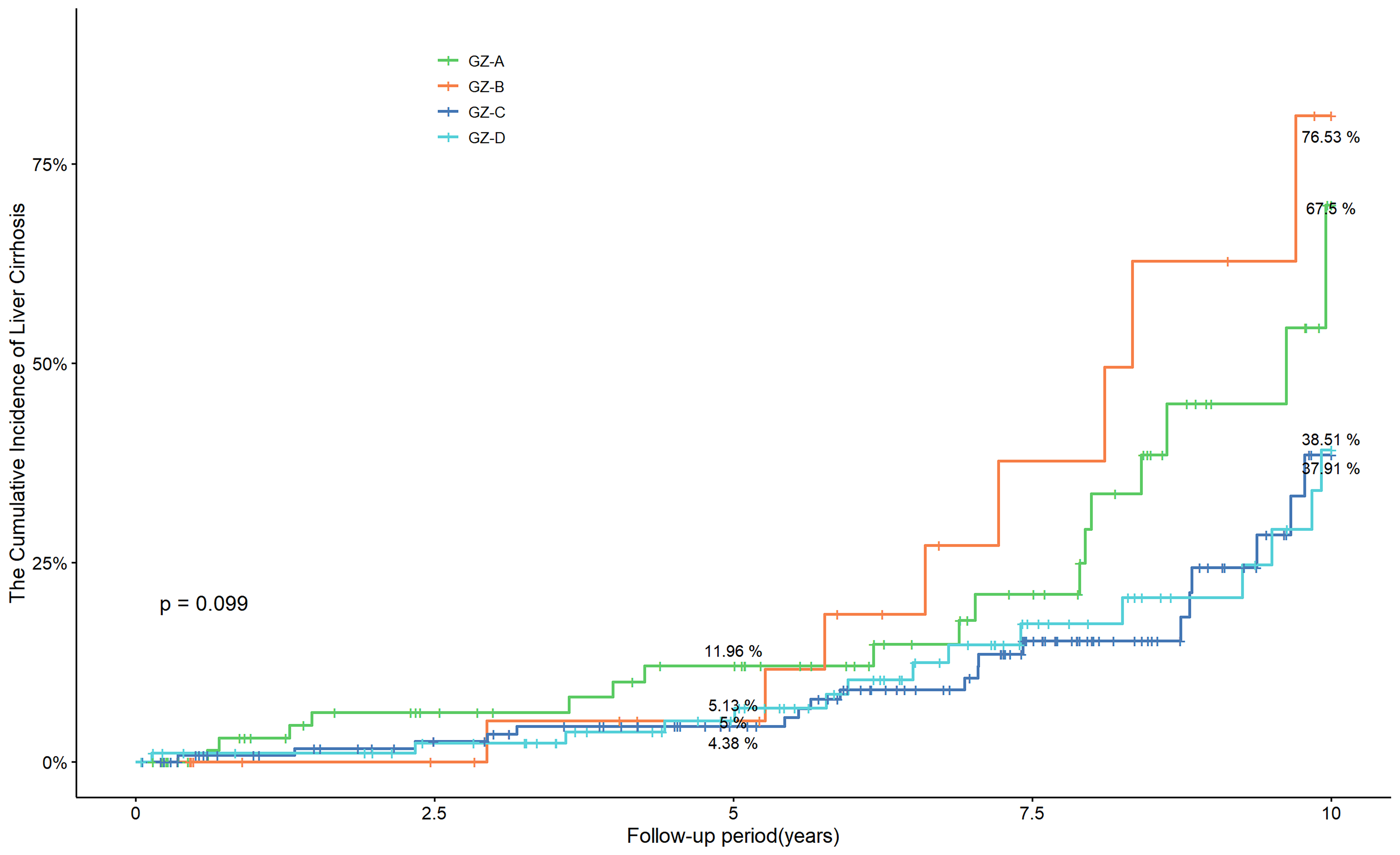

Supplement: Supplemental Material [file IANN_A_2399757_SM8435.zip › Suppl_Data/Supplementary Figure1(300dpi).tif]
